# Stroke Outcomes in Malawi, a Country with High Prevalence of HIV: A Prospective Follow-Up Study

**DOI:** 10.1371/journal.pone.0033765

**Published:** 2012-03-29

**Authors:** Terttu Heikinheimo, Daniel Chimbayo, Johnstone J. Kumwenda, Sam Kampondeni, Theresa J. Allain

**Affiliations:** 1 Department of Neurology, Helsinki University Central Hospital, Helsinki, Finland; 2 Department of Medicine, College of Medicine, Blantyre, Malawi; 3 Department of Radiology, College of Medicine, Blantyre, Malawi; Innsbruck Medical University, Austria

## Abstract

**Background:**

Stroke contributes significantly to disability and mortality in developing countries yet little is known about the determinants of stroke outcomes in such countries. 12% of Malawian adults have HIV/AIDS. It is not known whether having HIV-infection alters the outcome of stroke. The aim of this study was to document the functional outcome and mortality at 1 year of first-ever acute stroke in Malawi. Also to find out if the baseline variables, including HIV-infection, affect the outcome of stroke.

**Methods and Findings:**

147 adult patients with first-ever acute stroke were prospectively followed up for 12 months. Conventional risk factors and HIV-infection were assessed at baseline. Stroke severity was evaluated with modified National Institute of Health Stroke Scale (mNIHSS) and functional outcome with modified Rankin scale (mRS). Fifty (34%) of patients were HIV-seropositive. 53.4% of patients had a poor outcome (severe disability or death, mRS 4–6) at 1 year. Poor outcome was related to stroke severity and female gender but not to presence of HIV-infection. HIV-seropositive patients were younger and had less often common risk factors for stroke. They suffer more often ischemic stroke than HIV-seronegative patients.

**Conclusions:**

Mild stroke and male gender were associated with favourable outcome. HIV-infection is common in stroke patients in Malawi but does not worsen the outcome of stroke. However, it may be a risk factor for ischemic stroke for young people, who do not have the common stroke risk factors. Our results are significant, because stroke outcome in HIV-seropositive patients has not been studied before in a setting such as ours, with very limited resources and a high prevalence of HIV.

## Introduction

In developing countries the number of disability-adjusted life years caused by cerebrovascular disease is more than six times higher than in high-income countries: More than 85% of all stroke deaths occurs in low-income countries [1].

There is a paucity of data on the outcome of stroke in sub-Saharan Africa. Only two reports are available, both from West Africa. One-year case-fatality of stroke in the Gambia and Senegal was 62% and 50% respectively. In the Gambia hospital mortality following a stroke was 41% [2], [3]. Both of these countries have a relatively low HIV-1 prevalence. Malawi, like many of its neighbours, has a high HIV-1 prevalence. HIV-infected stroke patients present more often with cryptogenic strokes compared to HIV-seronegative individuals [4]–[6]. In a South African study, one third of HIV-1-infected patients, presenting with a stroke, had a recent or current opportunistic infection [7].

**Figure 1:**
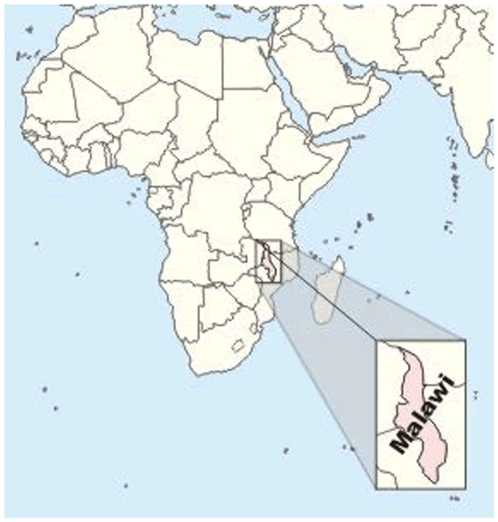
Map of Malawi in Africa. (source: http://www.un.org/depts/Cartographic/map/profile/malawi.pdf).

Malawi is situated in Central-Southern Africa (Figure 1). According to the Human Development Index, Malawi is among the countries with low human development, ranking 171 out of 187 countries [8]. Malawi has a population of 13.2 million. The life expectancy is 53 years for men and 54 years for women. Infectious diseases are still the main cause of mortality, but non-communicable diseases are estimated to account for 28% of all deaths [9]. 12% of the population aged 15–49 has HIV/AIDS. Mortality from stroke was over 7200 in 2002 [10]. A previous survey found that the seroprevalence of HIV among stroke inpatients at a central hospital in Malawi was 48% [4]. Malawi has a national programme for providing Highly Active Antiretroviral Treatment (HAART) free for HIV- infected patients. At the time of this study first-line treatment was a combination of three drugs: nevirapine, stavudine and lamivudine [11].

The primary aim of this study was to determine the functional outcome of first-ever acute stroke at one year of follow-up. Secondary aims were to describe the one-year mortality of stroke and to determine the effect of baseline demographics, including presence of HIV-infection.

## Methods

This was a prospective study of patients presenting with a first-ever acute stroke to the Queen Elizabeth Central Hospital (QECH) in Blantyre. QECH is the largest hospital in Malawi. It provides all levels of care, from primary to tertiary to patients within Southern Malawi. The QECH is the only public hospital within Blantyre District. Blantyre has a population of 1 million. It is the second largest city in Malawi after the capital city, Lilongwe (figure 1) [12].

The study recruitment took place between February 2008 and April 2009.

### Objectives

The objectives were to determine the functional outcome of first-ever acute stroke and to determine the effect of baseline demographics in Malawi, a sub-Saharan African country with high HIV-prevalence.

### Participants

Patients were eligible for the study if, they were 18 years or older, had a first-ever acute stroke (0–7 days before admission), and lived within 100 km of QECH. The diagnosis of stroke was based on clinical findings, according to WHO recommendations [13]. Patients with previous stroke-like symptoms, head trauma or other central nervous system (CNS) diagnoses were excluded from the study. Baseline assessment was carried out within 72 hours of admission.

### Description of Procedures or Investigations undertaken

Using information from the patient, their guardian and the patient's hand-held health record, demographic details, past medical history and medication use were recorded. If age was unknown it was estimated, within 5 years, by asking the patient's life history and age of their children. Current cigarette smoking and heavy alcohol use (estimated >100 g/day) were recorded. Study clinicians DC or TH carried out clinical examination. This included a detailed cardiovascular and neurological examination. The Mid Upper Arm Circumference (MUAC) was used to estimate nutritional status. Serum total, random cholesterol, full blood count, random or fasting capillary blood glucose (On Call Plus® glucometer), rapid plasma reagin-test to screen for syphilis (RPR), and presence of HIV-infection using two standard rapid immunoassays (Uni-Gold™ Recombigen® HIV and Determine® HIV-1/2) were measured. If the HIV-test was reactive the WHO HIV clinical stage was recorded [14] and serum CD4-count (FACS Count, Becton, Dickinson, San Jose, CA) was measured. Brain imaging was done by computed tomography-scan (CT, Philips single slice CT scanner, TOMOSCAN EG, Netherlands) or magnet resonance-imaging scan (MRI, 0.35T General Electric Signa Ovation excite with open configuration, China).

WHO definitions were used for hypertension, diabetes mellitus and hypercholesterolemia [15], [16]. MUAC≤23 cm was considered underweight and ≥33 cm obese [17].

Stroke severity at recruitment was assessed by the National Institute of Health Stroke Scale (NIHSS) [18]. This was modified; because people do not always know their age, the name of the current president of Malawi was asked instead (mNIHSS). mNIHSS 0–6 was considered mild, 7–12 moderate, 13–20 severe, ≥21 very severe stroke. The clinical subtype of stroke was recorded according to the classification of Bamford [19]. The modified Rankin scale (mRS) was evaluated from the history before the onset of stroke and by the clinical findings at the time of recruitment [20]: mRS 0–2 was considered good, mRS 3 fair, and 4–6 poor outcome.

Patients were followed up 6–8 weeks, six months and one year from stroke onset. At follow-up new health events, blood pressure and mRS were recorded. If possible, follow-up was done at a designated clinic at QECH. For patients who were unable to attend clinic a home visit was carried out. Alternatively the patients or their guardians were contacted by phone and the mRS was done based on information given.

### Ethics

The Malawi College of Medicine Research Ethical Committee (COMREC P 07/07/573) granted approval for the study. The patients, or, in case of reduced consciousness or severe dysphasia, the guardians, gave consent.

### Statistical methods

The primary outcome measure was mRS-score at one year, comparing a good/fair outcome (mRS 0–3) to a poor outcome (mRS 4–6). Data were analysed using Statview™ and STATA™ for multivariate analysis. Descriptive statistics (mean, SD) were used to describe the baseline data. Proportions were compared by X^2^ test and means by Student's t test, for normally distributed data, and Kruskal-Wallis test for non-parametric data.

## Results

**Figure 2:**
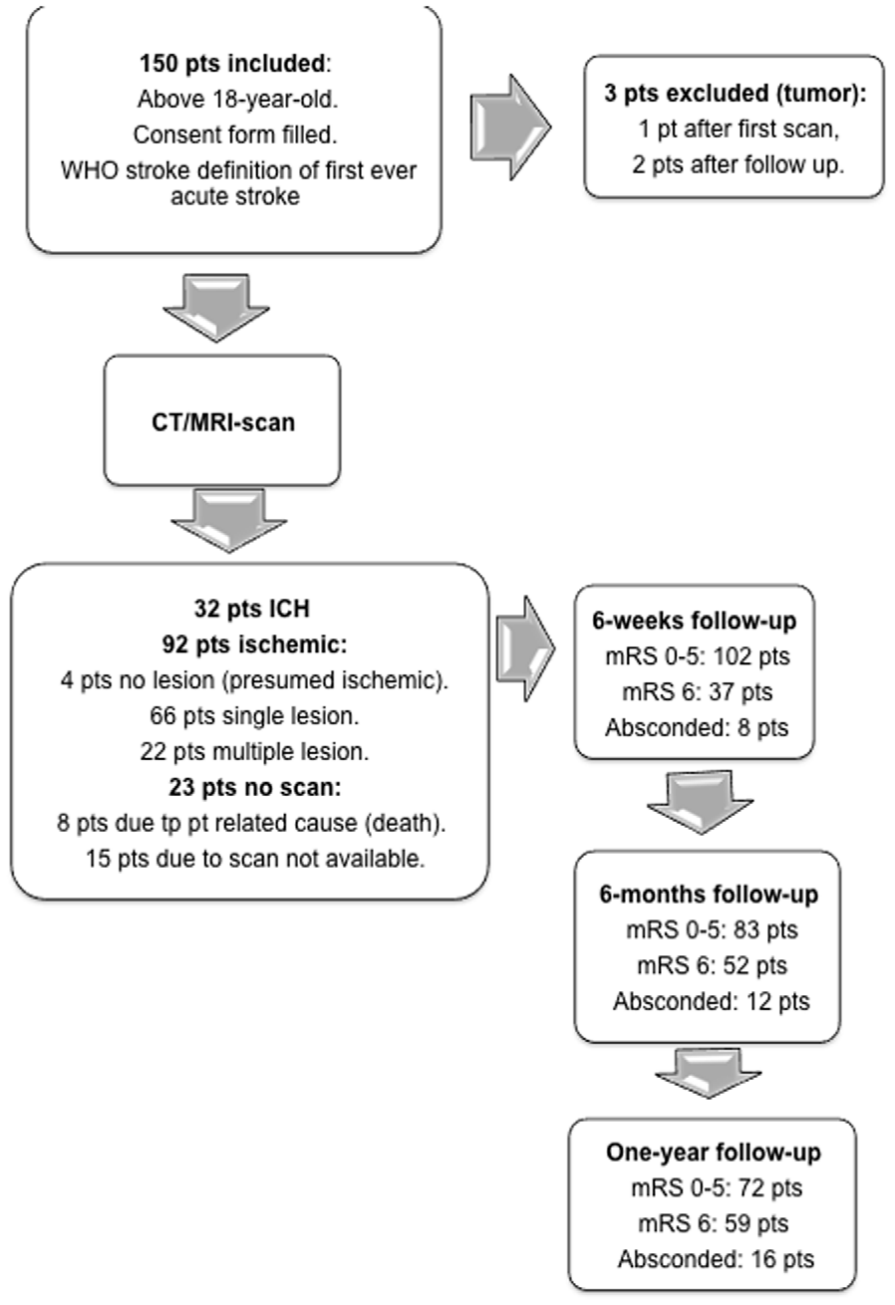
Study flow chart with CT/MRI-scan findings and outcome. pt/s = patient/s, WHO = world health organisation, CT/MRI = Computed tomography or magnetic resonance imaging, mRS = modified Rankin scale.

One hundred and fifty patients were recruited. Brain imaging was available in 127: 124 patients had CT and 3 had MRI. Three patients were excluded from analysis as their brain imaging or follow-up suggested probable malignancy, so the final sample was 147 (figure 2).

### Baseline characteristics (table 1) and etiological factors

**Table 1:** Baseline clinical features.

Catecory	Subcategory	HIV-negative N = 84	HIV-positive N = 50	HIV unknown N = 13	Total N = 147	P for comparison HIV-positive vs HIV-negative
**Age years (mean SD)**		61.9 (14.0)	39.8 (12.4)	63.6 (10.5)	54.2 (16.9)	<0.0001
**Female (%)**		40 (46.5)	26 (52.0)	5 (38.5)	71 (47.7)	0.54
**Hypertensive (n, %)**		61 (75.3)	12 (24.0)	10 (77.0)	84 (55.0)	<0.0001
**Diabetic (n, %)**		21 (25.0)	3 (6.0)	6 (46.2)	29 (19.7)	0.004
**Smoker (n, %)**		16 (19.3)	7 (14.0)	3 (23.1)	26 (17.7)	0.47
**Cholesterol >190 mg/dl (n, %)**		15 (17.9)	7 (14.0)	0	22 (15.0)	0.54
**Haemoglobin g/dL (mean SD)**		13.6 (2.5)	11.8 (5.8)	14.0 (2.8)	12.9 (2.6)	0.0003
**MUAC cm (n,%)**	**(mean SD)**	27.3 (4.2)	26.4 (3.8)	29.8 (5.2)	27.2 (4.2)	0.2
**(n, %)**	**≥33**	6 (7.1)	4 (8.0)	3 (23.1)	13 (8.8)	
	**24–32**	62 (73.8)	37 (74.0)	7 (53.8)	106 (72.1)	
	**≤23**	11 (13.1)	9 (18.0)	0	20 (13.6)	
	**not available**	5 (6.0)	0	3 (23.1)	8 (5.4)	
**Brain scan**	**IS** *	47 (55.6)	40 (80.0)	5 (38.5)	92 (62.6)	0.004
	**ICH**	25 (29.8)	5 (10.0)	2 (15.4)	32 (21.8)	
	**not scanned**	12 (14.3)	5 (10.0)	6 (46.2)	23 (15.6)	
**Clinical subtypes**	**TAC**	11 (13.1)	1 (2.0)	7 (53.8)	19 (12.9)	
	**PAC**	59 (70.2)	35 (70.0)	5 (38.5)	99 (67.3)	
	**LAC**	8 (9.5)	11 (22.0)	1 (7.7)	20 (13.6)	
	**POC**	6 (7.1)	3 (6.0)	0	9 (6.1)	
**Pre-stroke functional status**	**mRS 0–2**	83 (98.8)	47 (94.0)	13 (100.0)	143 (97.3)	0.46
	**mRS 3**	1 (1.2)	1 (2.0)	0	2 (1.4)	
	**mRS 4–5**	0	2 (4.0)	0	2 (1.4)	
**Stroke severity: n(%)**	**mNIHSS 0–6**	14 (16.6)	12 (24.0)	4 (30.8)	30 (20.4)	0.092
	**mNIHSS 7–12**	17 (20.2)	16 (32.0)	1 (7.7)	34 (23.1)	
	**mNIHSS 13–20**	24 (28.6)	11 (22.0)	1 (7.7)	36 (24.5)	
	**mNIHSS≥21**	29 (34.5)	11 (22.0)	7 (53.8)	47 (32.0)	

* Includes 4 subjects with normal scans.

IS = ischemic stroke, ICH = Intracerebral hemorrhage, MUAC = Mid Upper Arm Circumference, TAC = total anterior circulation stroke, PAC = partial anterior circulation stroke, LAC = lacunar stroke, POC = posterior circulation stroke.

The mean (±SD) age was 54.2 (±16.9) years, 47.7% were female. Hypertension, diabetes and hypercholesterolemia were present in 55.0%, 21.0% and 15.0%, respectively. Twenty-six (17.7%) admitted smoking tobacco and 22 (15.0%) were heavy alcohol drinkers. Fifty (34.0%) of patients were HIV-seropositive. They were significantly younger (39.8±12.4 vs 61.9±14.0 years, p<0.0001), had lower haemoglobin levels (11.8±5.8 vs 13.6±2.5 g/dL, p = 0.0003), and were less likely to have stroke risk factors of hypertension (75.3% vs 24.0%, p<0.0001), and diabetes (25.0% vs 6.0%, p = 0.004). There was a strong relationship between age at the time of first ever stroke, and HIV-status; 65.2% of those aged <55 years were HIV-seropositive whereas only 10.3% of those >55 years were HIV-seropositive (X^2^ = 43.1, p<0.0001). In those who were HIV-seropositive the WHO clinical staging was 1 or 2 in 39 (78.0%). Despite this, 28/35 in whom results were available, had CD4 cell counts of <300 cells/µl. Eleven subjects were taking HAART. Six had started HAART within the past 6 months, the remainder had been taking HAART for at least one year, however, three out of five patients with long term HAART use had CD4 <100 cells/µl, suggesting a possibility of HAART-failure. 100 patients had an ECG, of whom only 7 had atrial fibrillation (AF). The mean age of AF cases was 65.9±12 years and 4/7 were HIV-seropositive. Fifteen subjects had a reactive RPR suggesting syphilis infection, five of these were HIV-seropositive.

### Type of stroke, pre-stroke function and stroke severity

Thirty-two (32/124, 25.8%) scans showed intracerebral hemorrhage (ICH). The remainder were consistent with cerebral infarction (figure 2). ICH was associated with hypertension; 87.5% vs. 46.7% were hypertensive, (X^2^ = 15.4, p<0.0001) and was less common in HIV-seropositive patients (11.1% vs 34.7%, X^2^ = 8.1, p = 0.004). Multiple logistic regression controlling for age, gender and other vascular risk factors showed that hypertension and HIV-status were independently associated with the type of stroke; For ICH and hypertension (OR 6.7 95%CI 1.3–35.2, p = 0.024) for HIV-seropositive and ischemic stroke (OR 4.5 95%CI 1.03–20.4, p = 0.046). The clinical subtypes, pre-stroke functional status (mRS) and stroke severity (mNIHSS) are presented in table 1. The commonest stroke syndrome was partial anterior circulation (PACS). 36.1% had left hemiparesis. The vast majority of subjects were fully independent before their stroke and mRS did not vary with age, gender or HIV-status. On admission 83 (56.5%) had severe strokes (mNIHSS ≥13). Stroke severity was associated with older age (mNIHSS ≥13 59.2±15.5 vs 48.5±16.9 years, p<0.0001), but not with gender or HIV-status. In multiple logistic regression none of these variables were significantly, independently associated with stroke severity.

### Stroke outcomes

Thirty-three patients (22.4%) died in hospital. Outcome at 1-year was unavailable on 16 subjects. Cumulative mortality rates at 6 weeks, 6 months, and 1-year were 37/134 (27.6%), 52/134 (38.8%), and 59/131 (45.0%), (see table 2). Mortality at 1-year was significantly related to older age (58.5±16.6 vs. 54.1±15.4 years, p = 0.013), female gender (55% females vs 36.6% male, X^2^ = 4.4, p = 0.035) and stroke severity on admission (mNIHSS≥13, 60.3% mortality vs. mNIHSS 0–12, 22.6% mortality, X2 = 18.0, p<0.0001), but not HIV. High mNIHSS score at presentation (mNIHSS ≥13) did not relate to type of stroke, hemorrhagic or ischemic (X^2^ = 2, p = 0.15). In multiple logistic regression mNIHSS≥13 at presentation (OR 41 95%CI 1.2–14.3, p = 0.024) and female gender (OR 3.8 95%CI 1.4–10.3, p = 0.008) were independent determinants of mortality at one year.

**Table 2:** Functional outcome and mortality.

Category	Subcategory	HIV-seronegative N = 84	HIV-seropositive N = 50	HIV unknown N = 13	Total N = 147	P for comparison HIV positive vs HIV negative
**mRS at 6 weeks n(%)**	**mRS 0–2**	16 (19.0)	15 (30.0)	1 (7.7)	32 (21.8)	0.015
	**mRS 3**	14 (16.6)	12 (24.0)	4 (30.8)	30 (20.4)	
	**mRS 4–5**	27 (32.1)	7 (14.0)	0	34 (23.1)	
	**mRS 6**	20 (23.8)	9 (18.0)	8 (61.6)	37 (24.5)	
	**Cumulative deaths**	20	9	8	37	
	**Missing data**	7	7	0	14	
**mRS at 6 months n(%)**	**mRS 0–2**	29 (34.5)	17 (34.0)	2 (15.4)	48 (32.7)	0.58
	**mRS 3**	11 (13.1)	6 (12.0)	3 (23.1)	20 (13.6)	
	**mRS 4–5**	10 (11.9)	3 (6.0)	0	13 (8.8)	
	**mRS 6**	7 (8.3)	8 (16.0)	0	15 (10.2)	
	**Cumulative deaths**	27	17	8	52	
	**Missing data**	7	7	0	14	
**mRS at 1 year n(%)**	**mRS 0–2**	31 (36.9)	17 (34.0)	2 (15.4)	50 (34.0)	0.29
	**mRS 3**	3 (3.6)	6 (12.0)	2 (15.4)	11 (7.5)	
	**mRS 4–5**	10 (11.9)	1 (2.0)	0	11 (7.5)	
	**mRS 6**	5 (6.0)	1 (2.0)	1 (7.7)	7 (4.8)	
	**Cumulative deaths**	32	18	9	59	
	**Missing data**	8	8	0	16	

The functional outcome (mRS) at 6 weeks, 6 months and 1 year are shown in table 2. At one year 61/131 (46.6%) had mRS 0–3, and 70/131 (53.4%) had mRS 4–6. Poor outcome was associated with older age; (58.8±16.6 vs 52.6±15.2 years, p = 0.027), female gender; 62.3% of women vs 45.1% of men, (X^2^ = 3.9, p = 0.048), and more severe stroke, mNIHSS ≥13, at presentation (X^2^ = 35.9, p<0.0001), but not with HIV-status. In multiple logistic regression mNIHSS ≥13 at presentation (OR 4.5 95%CI 1.7–11.8, p = 0.002), and female gender (OR 2.3 95%CI 1.0–5.5, p = 0.05) were independent determinants of a poor outcome (mRS 4–6) at 1 year.

### Post-stroke care

The average length of hospital stay was 15.7±10.7 days. Twenty-nine (19.7%) patients were transferred to a residential rehabilitation unit. Nine (6.1%) patients received outpatient physiotherapy. 127 (86.4%) patients were put on ASA (75 mg once a day). In 20 of these subsequent CT revealed hemorrhage and the ASA was stopped. Anticoagulants, ACE-inhibitors and statins are not routinely available so were not considered in the evaluation. Thirteen patients had another non-fatal stroke during one-year follow-up. Two patients developed secondary epilepsy and one patient developed thalamic pain syndrome. One patient was diagnosed hypertensive during follow-up. Of the HIV-seropositive patients that were not on HAART previously, 11 started it during the follow-up year.

## Discussion

This study describes mortality and functional outcomes in a group of patients presenting with a first-ever acute stroke in a sub-Saharan African country with high HIV-prevalence. The prevalence of HIV-infection was 34%. We used a strict case definition to identify stroke patients and excluded non-stroke diagnoses on the basis of the clinical presentation and brain imaging.

Hypertension was the most prevalent vascular risk factor, and was present in 55% of patients. Diabetes was present in 21% of patients. Both of these risk factors were very much clustered in older patients. A recent survey in Malawian adults, aged 25–64 years, showed the prevalence of raised blood pressure, raised blood glucose and hypercholesterolemia were 31.6%, 5.6%, and 10.0% respectively [16]. Thus, the patients presenting with stroke at QECH, had a much higher prevalence of vascular risk factors than the general Malawian population, particularly hypertension and diabetes. Using MUAC as a marker of nutritional status, levels of obesity were similar to the general population, where 5.5% have a BMI ≥30 Kg/m^2^ [16]. Smoking and excessive drinking are well-known risk factors for stroke. In our study rates of smoking were lower than those reported in the general population [16]. The rate of AF in this population was low at 7%, although higher than that reported for black Africans with stroke in South Africa [21]. This may reflect a younger age group in the South African study where the average age was 51 years. Interestingly, although patients with AF were in the older age group, 4 out of 7 were HIV-seropositive, suggesting a possible effect of HIV on the ageing heart.

Fifty (34%) of patients were HIV-seropositive. The average age of these patients was 39.8 years. They had less often hypertension and diabetes and more often ischemic stroke. This supports the proposed suggestion that HIV is a risk factor for ischemic stroke [22], [23]. In many this was the first time they were aware of their HIV-status and there were no other clinical indicators of immunosupression. Twenty-two had CD4 counts <250 cells/µL and were not on HAART so were eligible to start HAART according to the Malawi National Programme [11]. Eleven patients started HAART during the period of follow-up. It is a concern that 11 patients (50%) that were eligible did not start HAART, possibly due to mobility problems or lack of understanding of the need. Of the eleven patients that were already on HAART prior the stroke, five had started HAART in the previous six months, raising the possibility that ischemic stroke could be part of an immune reconstitution inflammatory syndrome (IRIS) [24]. The numbers in our study are too small to allow firm conclusions, but this is an area, which warrants further investigation. Long term HAART use is associated with increased cardiovascular risk, which was possibly relevant in some of our patients [25]. In addition three out of the 5 patients on long term HAART had CD4 counts <100 cells/µL, suggesting possible immunological treatment failure. In these patients the stroke led to the diagnosis of possible treatment failure.

One-year outcome data was available on almost 90% of patients recruited. The main determinants of death, or death and severe disability at 1-year were severity of stroke on admission, and gender. A gender difference in favour of men in stroke outcome is known. In earlier reports this is reported to be due to women being older, having higher rates of AF, and being less likely to receive acute treatment [26]. These factors were not true in our study and there was no difference in severity of stroke between the genders, however, female patients still had a worse outcome. In Malawi, women play a key role as carers in vital issues like patient's food and fluid intake, hygiene, and mobilisation during the hospital stay, and at home. It may be that female patients are less likely to receive such care from their guardians. This and other socio-demographic factors may have an influence on why female stroke patients have a worse prognosis in Malawi, and other developing countries [2]. Differences in in-hospital, and 1-year mortality compared to the previous study from Malawi [4] and the studies from Gambia and Senegal may possibly reflect different recruitment criteria leading to differences in the baseline characteristics of the subjects, different patterns of co-morbidity, and possibly differences in post-stroke care [2], [3].

### Limitations

This study has a few shortcomings. Recruitment was hospital-based so does not include stroke patients with mild symptoms who do not present to hospital, or the very severest strokes, who die before coming to hospital, or are kept at home. At times we were frustrated by the limitations of our setting. Equipment, particularly the CT and ECG-machine broke and could not be repaired within the time frame of the study. Hence, there is some missing data in these areas.

### Conclusions

Patients presenting with stroke in central hospital in Malawi divided into two populations. Older patients, over 55 years, were most likely to be HIV-seronegative and have “traditional” vascular risk factors. Younger patients were very likely to be HIV-seropositive and vascular risk factors were uncommon. This suggests that HIV-infection could be a risk factor for stroke for young people without common stroke risk factors. All the patients received similar post-stroke care, and although there were differences in functional ability, as reflected by the mRS-score at presentation, the 1-year outcome was not associated to HIV-status. The 1-year mortality was 40.1%. 47.6% had a poor outcome (mRS 4–6). Stroke severity on admission and female gender were the only two independent factors associated with outcome. It is also a concern that only half of those with HIV who were eligible to start HAART did so, during one year of follow up.
